# 

**Published:** 1902-09

**Authors:** 


### Society Notes.

### Meeting of the Tri-State Medical Society.

The fourteenth annual meeting of the Tri-State Medical Society will be held at Birmingham, Ala., October 8, 9 and 10, 1902. This meeting promises to be of unusual interest from present indications. One of the prominent features of the last meeting, and which attracted considerable attention, was the discussion of sociological questions. The papers in this section excited considerable comment locally in many of the journals throughout the country.

